# Clinical and Demographic Characteristics of Families Attending the Epilepsy, Neuromuscular, and Child Wellbeing Clinics

**DOI:** 10.7759/cureus.43651

**Published:** 2023-08-17

**Authors:** Ahmed K Bamaga, Anas S Alyazidi, Tarek Z Arabi, Alaa Hamad, Dalal F Alageel

**Affiliations:** 1 Pediatrics, King Abdulaziz University Hospital, Jeddah, SAU; 2 Medicine, King Abdulaziz University Hospital, Jeddah, SAU; 3 Medicine, Alfaisal University, Riyadh, SAU

**Keywords:** parents, physician-patient relations, behavioral empathy, epilepsy, empathy

## Abstract

Background

Neurological diseases entail a broad spectrum of disorders. Among such ailments are epilepsy and neuromuscular disorders which impose a substantial burden on children and their families. Ensuring adequate access to outpatient services is crucial for these children regardless of the subclinical specialty, and clinicians can better comprehend the caregivers' perspectives by being aware of their backgrounds which can be aided using epidemiological studies.

Methods

In June 2023, a cross-sectional study was carried out in pediatric neurology clinics at a tertiary care center. The study included all families with a child or more (14 years and younger) diagnosed with neurological disorders. The study adopted a three-section survey delivered to participants recruited using a non-probability sampling technique to achieve a 95% confidence interval with a 5% margin of error.

Results

A total of 821 families participated in this study. The mean age of respondents was 40.46±8.72 years. Of the affected children, there were 600 (73.08%) children following up with the general neurology and epilepsy clinics, 164 (19.98%) were following up with the neuromuscular disorders clinics, and 57 (6.94%) were following up with the neurogenetic clinics. Familial status had no association with the type of clinic the patient was following up with *p*=0.0054. Single respondents had a significantly higher prevalence of children with epilepsy (*p*<0.0001). Parents with a high school level of education or lower had a significantly greater prevalence of epilepsy clinic follow-ups (*p*=0.0048).

Conclusion

The findings of this study contribute to the assessment of prevalent neurological disorders in children and shed light on the family dynamics surrounding these conditions. Through statistical analysis, the study establishes connections between certain demographic and clinical traits and specific neurological disorders among pediatric patients and their families. The study emphasizes the importance of socio-economic and socio-clinical support in promoting child health in such cases. Similar research would offer a more accurate portrayal of the challenges faced by families in these circumstances.

## Introduction

Neurological diseases entail a broad spectrum of disorders and pathologies that can arise from abnormalities in the structure, biochemical, brain electricity, spinal cord, cranial nerves, peripheral nerves, nerve roots, autonomic nervous system, neuromuscular junction, and muscles [1,2]. Abnormalities can also be a result of genetic disorders [3] that are prevalent among pediatric patients, especially in countries with higher consanguinity rates such as Saudi Arabia [4,5]. Among such ailments, epilepsy and neuromuscular disorders impose a substantial burden not only on the affected children but also on their caregivers [6]. In epilepsy clinics, it is common to encounter cases of generalized-onset tonic-clonic, focal-onset with or without impaired awareness, and focal to bilateral tonic-clonic seizures along with other epileptic conditions [7]. Such patients often experience co-morbid neuropsychological effects, migraines, and psychological problems [8]. However, in neuromuscular clinics, cases can include disorders of the anterior horn cell (e.g., spinal muscular atrophy), peripheral nerve (e.g., Charcot-Marie-Tooth), the neuromuscular junction (e.g., congenital myasthenic syndrome), and myopathies [9]. Despite the majority of these cases being of genetic etiology, many conditions require specialized neurogenetic clinics, whereas accurately genotyped patients can obtain a more prominent gain from therapeutic and prevention options [10]. Ensuring adequate access to outpatient services is crucial for these children regardless of the subclinical specialty, and clinicians can better comprehend the caregivers' perspectives by being aware of their backgrounds [11]. Moreover, healthcare professionals can advocate for measures to alleviate the financial burden imposed by these disabling diseases by gaining insight into the challenges faced by these families, as highlighted in epidemiological studies [8]. Several studies have evaluated the profile of patients treated in outpatient neurology clinics; however, there remains a dearth of research specifically focused on the pediatric population, resulting in limited literature in this domain. Therefore, this study aims to address this gap by providing a comprehensive description of the clinical and demographic patterns of pediatric patients attending general pediatric neurology, epilepsy, neuromuscular, and child wellbeing clinics at a tertiary care center in Jeddah, Saudi Arabia. Through this investigation, we seek to understand and inform effective strategies for managing and supporting pediatric patients with neurological conditions and their caregivers in this region.

## Materials and methods

Study design and setting

In June 2023, a cross-sectional study was carried out in the pediatric neurology clinics at King Abdulaziz University Hospital (KAUH) in Jeddah, Saudi Arabia. The study utilized an online survey format, which was distributed among families attending the clinics. To reach the participants, patients' phone numbers were retrieved from the electronic medical records and the health information system. The study adhered to the Strengthening the Reporting of Observational Studies in Epidemiology (STROBE) reporting guideline for cross-sectional studies [12]. This study followed the ethical guidelines for cross-sectional studies, reviewed and approved by the Unit of Biomedical Research Ethics at King Abdulaziz University with reference number (273-23). The study was conducted according to the World Medical Association Declaration of Helsinki, and informed consent was waived due to the nature of the study design. All revealing data were masked, and patients' privacy was ensured throughout the conduct of the study.

Study population

The study included all families with a child or more (14 years and younger) diagnosed with neurological disorders and with prior admission or who are currently following up at KAUH outpatient child neurology clinics. Ages 14 years and younger were determined as pediatrics according to our hospital policy. The clinics include the general pediatric neurology clinic, epilepsy clinic, neuromuscular clinic, and child wellbeing clinic. Families visiting the clinics from 2019 to June 2023 were enrolled in the study.

Sampling methodology

The survey comprised three sections that included a consent statement, demographic data, and a clinical profile. The participants were only able to proceed with the survey after granting their consent in the first section. The demographic data section entailed a mixture of demographic-based questions such as age, gender, and marital status, in addition to questions about the patient’s disease and family history. The clinical profile section included a thorough assessment of their clinical status, their medical history, and the presence of any inherited or genetic conditions. Subsequently, the collected data was automatically entered into a database. The appropriate sample size was determined after calculating the population size and employing Raosoft Sample Size Calculator software [13] using a non-probability sampling technique to achieve a 95% confidence interval (CI) with a 5% margin of error.

Data analysis

Statistical analysis was carried out using Statistical Package for the Social Sciences (SPSS) version 26 (IBM SPSS Statistics, Armonk, NY, USA) with a statistical significance of p<0.05 and 95% confidence intervals. Measures of central tendency were calculated to describe quantitative variables. Frequencies and percentages were used for categorical variables. Categorical variables were compared using the Chi-square test. One-way analysis of variance (ANOVA) was used to analyze statistical differences among the variables.

## Results

Demographic characteristics 

Among the 821 families who participated in our study, the mean age of respondents was 40.46±8.72 years. The majority of respondents (64.43%) were fathers, 88.19% were married, and all were Muslim. Approximately half (51.16%) of the participants achieved a bachelor’s degree or higher, 76.13% were Saudi, and 78.81% resided in Jeddah. About 96.59% of participants reported that they lived in the same house as their children. Of the affected children, there were 600 (73.08%) children following up with the general neurology and epilepsy clinics, 164 (19.98%) were following up with the neuromuscular disorders clinics, and 57 (6.94%) were following up with the neurogenetic clinics. Concerning household income, participants disclosed various income levels, with 27.04% reporting an income of 10,001-15,000 Saudi Arabian Riyals (SAR), 18.78% reporting 3,000 SAR, 14.98% reporting 3,001-5,000 SAR, and 12.79% reporting 7,501-10,000 SAR. Despite that the majority of participants reported that they do not live with their parents (63.22%); of those, 96.59% reported living with their children. However, slightly more than half of the participants (57.86%) reported that their parents rely financially on them. The majority live in Jeddah (78.81%) where the study was held, and similarly, the majority live in an apartment (81.24%). As for their clinical status, the vast majority (95.98%) have one to three children following up in the clinics with 68.94% regularly following up with a specialized neurologist. Further characteristics of our patients can be found in Table 1.

**Table 1:** Demographic characteristics of patients attending the clinics. Data includes clinical background of participants.

Parameter	N(%)/mean±SD
Age	40.46±8.72
Gender	
Male	529 (64.43%)
Female	292 (36.57%)
Marital status	
Married	724 (88.19%)
Single	85 (10.35%)
Widow	12 (1.46%)
Educational level	
High school and below	307 (37.39%)
Diploma	84 (10.23%)
Bachelor’s degree and above	420 (51.16%)
Nationality	
Saudi	625 (76.13%)
Non-Saudi	196 (23.87%)
Current job	
Government sector	292 (35.57%)
Housewife	207 (25.21%)
Military sector	69 (8.40%)
Private sector	163 (19.85%)
Retired	39 (4.75%)
Student	15 (1.83%)
Unemployed	36 (4.38%)
Monthly family income (SAR)	
<3,000	154 (18.78%)
3,001-5,000	123 (14.98%)
5,001-7,500	78 (9.50%)
7,501-10,000	105 (12.79%)
10,001-15,000	222 (27.04%)
15,001-20,000	97 (11.81%)
>20,001	42 (5.12%)
Do you live in the same house with your parents?	
Yes	302 (36.78%)
No	519 (63.22%)
Do you live in the same house with your children?	
Yes	793 (96.59%)
No	28 (3.41%)
Do your parents rely on you financially?	
Yes	346 (42.14%)
No	475 (57.86%)
City of living	
Jeddah	647 (78.81%)
Makkah	72 (8.77%)
Riyadh	3 (0.37%)
Others	99 (12.06%)
Type of accommodation	
Apartment	667 (81.24%)
Social housing	9 (1.10%)
Villa	145 (17.66%)
Ownership of accommodation	
Rental	511 (62.24%)
Owned	307 (37.39%)
Government housing	3 (0.37%)
Do you live in the same city as your parents?	
Yes	563 (68.57%)
No	258 (31.43%)
Are you diagnosed with any illnesses?	
Yes	180 (21.92%)
No	641 (78.08%)
What type of disease does your child have?	
Neuromuscular disorder	164 (19.98%)
Genetic	57 (6.94%)
Epilepsy	600 (73.08%)
Number of children following up	
1-3	788 (95.98%)
4-6	24 (2.92%)
>8	9 (1.10%)
Do you regularly follow up with a specialized neurologist?	
Yes	566 (68.94%)
No	255 (31.06%)
Do you have health insurance?	
Yes	141 (17.17%)
No	680 (82.83%)

Bivariate assessment of the clinical status and demographic data

Familial status had no association with the type of clinic the patient was following up with *p*=0.0054 (Table 2). Single respondents had a significantly higher prevalence of children with epilepsy (*p*<0.0001). Parents with a high school level of education or lower had a significantly greater prevalence of epilepsy clinic follow-ups (*p*=0.0048). Nationality had no impact on the type of clinic (*p*=0.3628). Families receiving the lowest category of monthly income had increased rates of epilepsy (*p*<0.0001). Type of accommodation, presence of deceased children, and regular follow-up with a neurologist had no impact on the type of clinic (Table 2).

**Table 2:** Bivariate analysis of the type of attendant clinic and characteristics of participants. The asterisk sign (*) indicates a statistically significant relationship (p<0.05).

Parameter	Epilepsy	Neuromuscular disorders	Genetic disorders	*P*-value
Familial status				
Mother	208 (71.23%)	72 (24.66%)	12 (4.11%)	0.0054*
Father	392 (74.10%)	92 (17.39%)	45 (8.51%)	
Marital status				
Married	518 (71.55%)	54 (7.46%)	152 (20.99%)	<0.0001*
Single	76 (89.41%)	3 (3.53%)	6 (7.90%)	
Widowed	6 (50.00%)	6 (50.00%)	0 (0.00%)	
Level of education				
High school and below	232 (75.57%)	54 (17.59%)	21 (6.84%)	0.0048*
Diploma	48 (57.14%)	24 (28.57%)	12 (14.29%)	
Bachelor’s degree and above	320 (74.42%)	86 (20.00%)	24 (5.58%)	
Nationality				
Saudi	461 (73.76%)	125 (20.00%)	39 (6.24%)	0.3628
Non-Saudi	139 (70.92%)	39 (19.90%)	18 (9.18%)	
Current job				
Employed	396 (75.57%)	92 (17.56%)	36 (6.87%)	0.0038*
Housewife	135 (6.52%)	60 (29.00%)	12 (5.80%)	
Retired	27 (69.23%)	9 (23.08%)	3 (7.69%)	
Student	12 (80.00%)	0 (0.00%)	3 (20.00%)	
Unemployed	30 (83.33%)	3 (8.33%)	3 (8.33%)	
Monthly income (SAR)				
<7,500	265 (74.65%)	63 (17.75%)	27 (7.61%)	<0.0001*
7,501-15,000	240 (73.39%)	57 (17.43%)	30 (9.17%)	
>15,001	95 (68.35%)	44 (31.65%)	0 (0.00%)	
City of living				
Jeddah	478 (73.88%)	121 (18.70%)	48 (7.42%)	0.0129*
Mecca	47 (65.28%)	25 (34.72%)	0 (0.00%)	
Riyadh	3 (100.00%)	0 (0.00%)	0 (0.00%)	
Others	72 (72.73%)	18 (18.18%)	9 (9.09%)	
Type of accommodation				
Apartment	486 (72.86%)	130 (19.49%)	51 (7.65%)	0.1712
Social housing	9 (100.00%)	0 (0.00%)	0 (0.00%)	
Villa	105 (72.41%)	34 (23.45%)	6 (4.14%)	
Ownership of accommodation				
Rental	391 (76.52%)	87 (17.03%)	33 (6.46%)	0.0375*
Owned	206 (67.10%)	77 (25.08%)	24 (7.82%)	
Government housing	3 (100.00%)	0 (0.00%)	0 (0.00%)	
Number of children following up at the neurology clinic				
1-3	567 (71.95%)	164 (20.81%)	57 (7.23%)	0.0130*
4-6	24 (100.00%)	0 (0.00%)	0 (0.00%)	
>7	9 (100.00%)	0 (0.00%)	0 (0.00%)	
Do you regularly follow up with a neurologist?				
Yes	409 (72.26%)	121 (21.38%)	36 (6.36%)	0.2027
No	191 (75.20%)	42 (16.54%)	21 (8.27%)	
Do you have health insurance for your sick children?				
Yes	96 (68.09%)	27 (19.15%)	18 (12.77%)	0.0114*
No	504 (74.12%)	137 (20.15%)	39 (5.74%)	
Do you have deceased children?				
Yes	118 (67.82%)	44 (25.29%)	12 (6.90%)	0.1383
No	482 (74.50%)	120 (18.55%)	45 (6.96%)	

## Discussion

**Figure 1:**
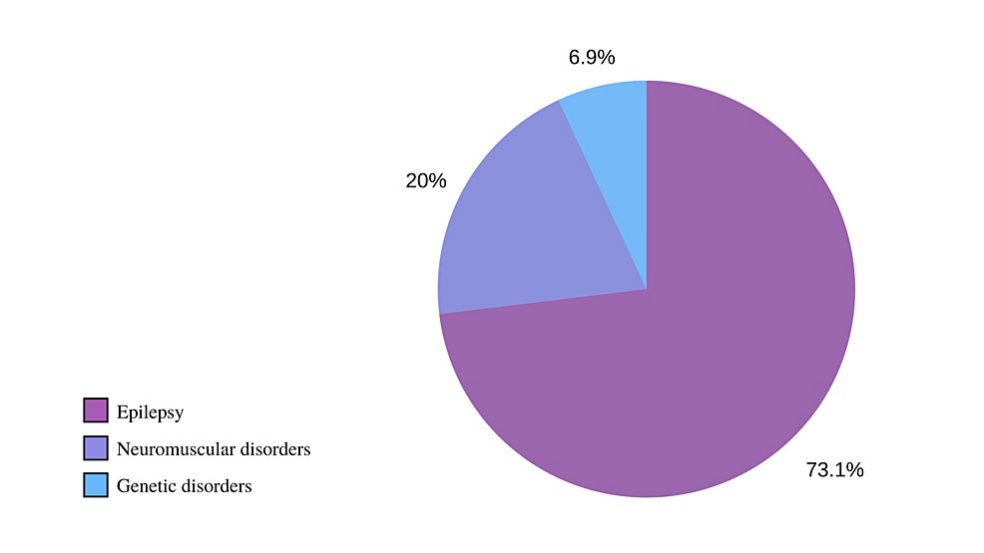
The distribution of participants according to the reported disorders.

The findings of this study provide a comprehensive perspective on the pattern and epidemiology of families attending pediatric neurology clinics. Despite the prevalence of neurological disorders among children, their impact is often underestimated in certain countries [3], and the reality of those family is extremely rare to be presented in the literature. Notably, the study revealed that the majority of participants and caregivers were fathers, challenging the historical perception that mothers solely bear the responsibility of childcare. Several studies have highlighted the increasingly prominent roles of fathers, especially in healthcare decision-making for their families and offspring [14-16]. Moreover, this study aligns with the growing recognition that positive involvement from both parents contributes to child health and development [14,17]. The majority of participants (88.19%) were married, indicating the potential benefits of a stable family environment on child wellbeing [18]. This is aided by prior studies which indicated that children living with unmarried parents do not fare as well as children living with married parents [19,20]. It is noteworthy that most participants were employees, although surprisingly only a small percentage reported having any form of health insurance. Having healthcare insurance can significantly improve accessibility to the healthcare system, thereby positively impacting the health of children, families, and society at large. However, strikingly only 17.17% reported having health insurance of any form. Healthcare insurance despite their financial burden can be determinant in advancing accessibility to the healthcare system, whereas subsequently the health of children themselves, their families, as well as society at large can improve [21]. Various factors discussed earlier in the study have wide-ranging effects on child outcomes, spanning cognitive, behavioral, and health domains. Additionally, the study established significant associations between specific clinics and demographic and clinical characteristics. For instance, single/unmarried parents were more significantly associated with visiting clinics for children affected by epilepsy, likely due to the heightened level of support and commitment required in such cases. An inverse relationship between income and attendance at epilepsy clinics was also observed, in line with the well-documented connection between lower income and chronic neurological diseases. An association between lower income and higher attendance for epilepsy clinics was also established echoing the inverse relationship between income and chronic neurological diseases [22]. Also, epilepsy remains to be the leading neurological complaint [23] similarly found in our study with nearly 73.08% of patients being affected with some form of epilepsy (Figure 1). Such findings are echoed throughout the literature. A specific study included a large cohort (n=17,176) of children that also concluded similar findings of wider epilepsy prevalence [1]. In the context of global literature, our study presented novel data on underrepresented and vulnerable populations. The heterogeneity of findings continues to oppose an issue that requires additional research studies with broader and more comprehensive representation. In spite of these limitations, our study shed new light on the association of clinical and demographic characteristics with certain neurological disorders.

## Conclusions

The findings of this study contribute to the assessment of prevalent neurological disorders in children and shed light on the family dynamics surrounding these conditions. Through statistical analysis, the study establishes connections between certain demographic and clinical traits and specific neurological disorders among pediatric patients and their families. Notably, epilepsy remains the most prevalent neurological disorder compared to others. The study also uncovers a diversity of findings in the backgrounds of the families, indicating a range of factors that may influence the experience of neurological disorders in children. Moreover, it emphasizes the importance of socio-economic and socio-clinical support in promoting child health in such cases. To gain a more comprehensive understanding of the family realities and neurological disorders in pediatric patients, larger and more comprehensive studies, preferably conducted across multiple centers, are deemed necessary. Such research would offer a more accurate portrayal of the challenges faced by families in these circumstances.
